# Tau seeding activity begins in the transentorhinal/entorhinal regions and anticipates phospho-tau pathology in Alzheimer’s disease and PART

**DOI:** 10.1007/s00401-018-1855-6

**Published:** 2018-05-11

**Authors:** Sarah K. Kaufman, Kelly Del Tredici, Talitha L. Thomas, Heiko Braak, Marc I. Diamond

**Affiliations:** 1 Center for Alzheimer’s and Neurodegenerative Diseases, NL10.120, Peter O’Donnell Jr. Brain Institute, University of Texas Southwestern Medical Center, 6000 Harry Hines Blvd., Dallas, TX 75390 USA; 2 Graduate Program in Neuroscience, Washington University in St. Louis, St. Louis, MO USA; 3 Clinical Neuroanatomy Section/Department of Neurology, Center for Biomedical Research, University of Ulm, Ulm, Germany

**Keywords:** Alzheimer’s disease, FRET biosensor, Neurofibrillary tangles, Prion propagation, Tau seeding activity, Tau staging

## Abstract

Alzheimer’s disease (AD) is characterized by accumulation of tau neurofibrillary tangles (NFTs) and, according to the prion model, transcellular propagation of pathological “seeds” may underlie its progression. Staging of NFT pathology with phospho-tau antibody is useful to classify AD and primary age-related tauopathy (PART) cases. The locus coeruleus (LC) shows the earliest phospho-tau signal, whereas other studies suggest that pathology begins in the transentorhinal/entorhinal cortices (TRE/EC). The relationship of tau seeding activity, phospho-tau pathology, and progression of neurodegeneration remains obscure. Consequently, we employed an established cellular biosensor assay to quantify tau seeding activity in fixed human tissue, in parallel with AT8 phospho-tau staining of immediately adjacent sections. We studied four brain regions from each of *n* = 247 individuals across a range of disease stages. We detected the earliest and most robust seeding activity in the TRE/EC. The LC did not uniformly exhibit seeding activity until later NFT stages. We also detected seeding activity in the superior temporal gyrus (STG) and primary visual cortex (VC) at stages before NFTs and/or AT8-immunopositivity were detectable. AD and putative PART cases exhibited similar patterns of seeding activity that anticipated histopathology across all NFT stages. Our findings are consistent with the prion model and suggest that pathological seeding activity begins in the TRE/EC rather than in the LC. In the analysis of tauopathy, quantification of seeding activity may offer an important addition to classical histopathology.

**Electronic supplementary material:**

The online version of this article (10.1007/s00401-018-1855-6) contains supplementary material, which is available to authorized users.

## Introduction

Tauopathies constitute a diverse group of neurodegenerative diseases that include Alzheimer’s disease (AD). They are defined by the deposition of aggregated phospho-tau protein in the central nervous system [13, 31]. Tau aggregation is directly linked to the pathogenesis of tauopathies, as tau mutations that increase the propensity of tau to aggregate cause dominantly inherited dementia [2]. The neuropathology of AD, the most common form of dementia, features intraneuronal pretangle and neurofibrillary tangle (NFT) tau pathology as well as extraneuronal ghost tangles and various forms of extracellular amyloid beta (Aβ) plaques. This tau pathology has a characteristic regional pattern of progression, thereby permitting the distinction of different stages in asymptomatic and symptomatic individuals [4, 8]. Recently, it was proposed that early NFT stages with pathological changes confined to the anteromedial temporal cortex, and minimal or no Aβ deposits, may constitute a primary age-related tauopathy (PART) [11], a hypothesis that remains a source of debate [15]. The spatiotemporal pattern of tau pathology in AD correlates well with brain atrophy and cognitive decline observed in subjects [5, 20, 23]. Based on extensive experimental data, we and others have proposed that transcellular propagation of tau protein “seeds,” in the manner of prions, could underlie the inexorable spread of pathology in tauopathies [39].

Tau aggregates that accumulate in tauopathies exhibit a high degree of phosphorylation [31]. Traditional immunohistochemistry (IHC) has been the gold standard for disease staging and discrimination among tauopathy syndromes [1, 25, 30]. The monoclonal antibody AT8, which recognizes phospho-serine 202 and phospho-threonine 205 on aggregated tau protein, is a principal tool to define AD intraneuronal pathology (pretangles and neurofibrillary tangles) [33]. The AT8 signal increases with disease progression (Suppl. Fig. 1a–c) [8]. It first appears in the locus coeruleus (LC), and thereafter in a few additional brainstem nuclei with diffuse cortical projections (subcortical pretangle stages a–c). The first cortical lesions have been observed in neuronal processes (cortical pretangle stage 1a) and in projection neurons (cortical pretangle stage 1b) of the transentorhinal region (TRE) in the absence of Aβ deposits [9]. This led to the idea that tau aggregation in the LC may represent the earliest phase of AD pathogenesis [9, 16]. At neurofibrillary tangle (NFT) stage I, AT8 and Gallyas silver staining reveal neurofibrillary lesions restricted to selected brainstem nuclei and the TRE. Pathology then develops in the entorhinal cortex (EC) of the parahippocampal gyrus at NFT stage II. At NFT stage III, it begins to involve the CA1 sector of the hippocampal formation and enters the neocortical regions of the temporal neocortex adjoining the TRE. NFT stages IV and V are characterized by increasingly abundant tau pathology in neocortical regions. The superior temporal gyrus (STG, Brodmann Area 22) becomes involved at NFT stage V, and during NFT stage VI the primary neocortical areas, such as the primary visual field (VC, Brodmann Area 17), exhibit tau lesions [5, 8] (Suppl. Table 1). In comparisons of pathology and clinical presentation, over half of the subjects at NFT stages III–IV exhibited signs of mild cognitive impairment, and over 90% of subjects at NFT stages V–VI exhibited moderate to severe dementia [25].

The progressive accumulation of tau pathology in AD has long been recognized to involve neural networks [4, 6]. Recent work in vitro [17] and in vivo [10, 26, 28, 36, 38] indicates that in experimental systems tau assemblies (seeds) spread pathology between interconnected neurons and progressively trigger further aggregation of native tau. This is similar to the pathophysiology of prion diseases, where prion protein (PrP) adopts a beta sheet-rich conformation that self-assembles and acts as a template to convert native PrP to a pathogenic form [35, 37]. In general, transcellular propagation of aggregation appears to be a common feature of various proteins implicated in neurodegenerative diseases [12, 17, 32, 34, 36].

The term “prion” is controversial as applied to noninfectious neurodegenerative diseases [21, 22, 27, 44, 45]. We use it here to encompass the myriad of proteins that can shift conformation from a monomer to a self-replicating assembly that specifies biological activity based on its conformation [39]. Based on the prion hypothesis, we have hypothesized that tau seeding activity will mark incipient, submicroscopic protein aggregation before the occurrence of tau pathology that is visible by light microscopy.

We have previously developed a sensitive and specific cell-based “biosensor” assay to detect tau seeding activity in biological samples [18, 24]. When we used this assay in a transgenic mouse model of tauopathy, we observed seeding activity far in advance of detectable histopathology or accumulation of insoluble tau protein [24]. In fresh frozen tissue from AD subjects, we have also observed seeding activity in advance of predicted neuropathological changes [19]. However, in such studies fresh frozen samples are more difficult to obtain than fixed brain tissue, and do not allow direct anatomical comparison of seeding activity with high quality histopathology. To resolve this problem, we recently developed a method to quantify tau seeding activity in fixed, archived human brain sections [29]. This has allowed simultaneous AT8 IHC and measurement of seeding activity in fixed tissues classified as AD and PART, and in asymptomatic individuals. We have now assessed the relationship of seeding to phospho-tau pathology in the LC and in more distant cortical regions, thereby addressing fundamental questions about AD pathogenesis.

## Methods

### Culture of biosensor cells

Seeding assays were performed with a previously published biosensor cell line that stably express tau-RD(P301S)-CFP and tau-RD(P301S)-YFP (ATCC CRL-3275) [24]. All HEK293 cells were grown in complete media: Dulbecco’s Modified Eagle’s Medium (DMEM) (Gibco) with 10% fetal bovine serum (Sigma) and 1% penicillin/streptomycin (Gibco). Cells were cultured and passaged at 37 °C, 5% CO_2_, in a humidified incubator. Dulbecco’s phosphate buffered saline (Life Technologies) was used for washing the cells prior to harvesting with 0.05% Trypsin–EDTA (Life Technologies).

### Tau KO mouse breeding

To determine a true negative control tissue for assays, we used tau KO mice containing a GFP-encoding cDNA integrated into exon 1 of the MAPT gene. These were obtained from the Jackson Laboratory and maintained on a C57BL/6J background [42]. Animals were housed on a 12 h light/dark cycle and provided with food and water ad libitum. All animal maintenance and experiments adhered to the University of Texas Southwestern animal care and use protocol.

### Mouse sample collection and preparation

Animals were anesthetized with isoflurane and perfused with chilled PBS with 0.03% heparin. Whole-brains were drop-fixed in 4% paraformaldehyde in PBS overnight at 4 °C. Brains were incubated in 30% sucrose before sectioning. Sections were collected to equivalent volume of human samples (100 μm thickness × 4 mm circular punch biopsy) and placed in TBS with protease inhibitors (Sigma Aldrich complete protease inhibitor, EDTA free) as described below. Mouse and human samples were subsequently prepared in an identical fashion.

### Human sample staging and preparation

Human autopsy tissue used for this study was obtained from *n* = 247 individuals with AT8-positive tau pathology (116 females, 131 males, age range 14–97 years, Table 1) and 6 controls (4 females, 2 males, age range 45–72 years, Suppl. Table 2) in compliance with ethics committee guidelines at the University of Ulm as well as German federal and state law governing human tissue usage. The brain specimens included cases from university-affiliated hospitals in Germany. The brains were fixed in a 4% buffered aqueous solution of formaldehyde and subsequently archived for up to 25 years. Tissue blocks were excised and embedded in polyethylene glycol (PEG 1000, Merck, Carl Roth Ltd, Karlsruhe, Germany), and 100 μm sections were collected as previously described [8].

**Table 1:** Summary of *n* = 247 case samples

Tau stage	*N*	Female	Male	Aβ phase	Average age	Age range
Definite primary age-related tauopathy (PART)						
1b	24	11	13	0.0	39	14–55
I	32	12	20	0.0	48	21–66
II	40	25	15	0.0	65	41–83
III	27	10	17	0.0	78	61–94
IV	11	4	7	0.0	83	66–94
Alzheimer’s disease (AD)						
I	3	3	0	1.0	63	55–72
II	17	5	12	1.9	72	45–85
III	30	15	15	2.1	80	61–93
IV	24	15	9	2.6	83	68–94
V	26	15	11	3.3	80	62–97
VI	13	7	6	3.9	81	70–90

Neuropathological staging and disease classification were performed according to a previously published protocol [9] by H.B. after AT8 immunostaining using a monoclonal antibody PHF-Tau [1:2000; Clone AT8; Pierce Biotechnology, Rockford, IL, USA (Thermo Scientific)] for recognition of phosphorylated tau protein in non-argyrophilic pretangle material and in argyrophilic NFTs of the Alzheimer type. AT8 IHC visualizes the broadest spectrum of intraneuronal pathological tau: pretangles, NFTs, neuropil threads (NTs) in dendritic processes, somatic aggregates, and, notably, axonal aggregates. By contrast, Gallyas silver-iodide staining visualizes argyrophilic NTs and NFTs but not the aggregates in axons. Ghost tangles are extraneuronal lesions (‘tombstones’) that display weak staining with the Gallyas method and strong staining with the Campbell-Switzer silver-pyridine method. In contrast to both of these methods, AT8 IHC visualizes ghost tangles less effectively than Gallyas silver staining or not at all. The character and relative merits of thioflavin-S staining, Gallyas and Campbell-Switzer silver staining, as well as more conventional silver methods (the modified Bielschowsky and the Bodian methods) in relation to tau isoforms and to IHC have been discussed in detail elsewhere [40, 43]. Aβ deposition was staged using the monoclonal anti-Aβ antibody 4G8 (1:5000; Covance, Dedham, MA, USA) as recommended previously [25, 41]. PART classification included cases with tau stages 1b-IV, Aβ phase 0 (“definite PART”). AD classification included cases with tau stages 1b-VI, Aβ phase ≥ 1. Subjects that met the criteria for “possible PART” (Aβ phases 1–2) were included with the remainder of AD subjects, given the presence of concomitant tau and Aβ pathology in these individuals [11].

In the present study, 18 cases displayed coincident argyrophilic grain disease (AGD). Care was taken to exclude other non-AD tauopathies, including progressive supranuclear palsy, Pick’s disease, and corticobasal degeneration (Suppl. Tables 3 and 4). In addition, all cases were also immunostained and staged for sporadic Parkinson’s disease (PD), as described elsewhere [7]. A total of 18 cases showed coincident α-synuclein-positive Lewy pathology (Suppl. Tables 3 and 4). Two cases displayed coincident AGD and Lewy pathology.

From each case, including negative controls, 4 mm punch biopsies were collected by K.D.T. from unstained sections of the locus coeruleus (LC); the transentorhinal cortex (TRE) and entorhinal cortex (EC) (two separate adjacent punches were taken from this combined region, termed TRE/EC, for seeding analyses); the superior temporal gyrus (STG); and the primary visual cortex (VC) [8] with a punch biopsy tool (Kai Industries Co, Ltd. Japan) (Suppl. Fig. 2a–f). To avoid cross contamination of seeding activity between individuals and regions, punch biopsy tools were used only once for each sample. Samples were encoded and all subsequent preparation and seeding assays were performed in a blinded fashion. Tissue punches were stored in 1× TBS at 4 °C until use. Samples were transferred to 100 μL of 1× TBS with protease inhibitors (Sigma Aldrich complete protease inhibitor, EDTA free), and water-bath sonicated in PCR tubes for 120 min under 50% power at 4 °C (Qsonica Q700 power supply, 431MPX microplate horn, with chiller).

### Transduction of biosensor cell lines

Biosensor cells were plated at 25,000 cells per well in 96-well plates. After 18 h, cells were transduced with human tissue homogenates as previously described [32, 35]. Samples were added to Opti-MEM (Thermo Fisher Scientific) and incubated for 5 min (3.3 μL lysate with 6.7 μL of Opti-MEM per well). Lipofectamine was incubated with Opti-MEM (1.25 μL Lipofectamine with 8.75 μL Opti-MEM per well) for five minutes. Lipofectamine complexes were then mixed with samples and incubated for 20 min prior to addition to biosensor cells. Samples were assessed in triplicate. Cells were kept at 37 °C in a humidified incubator for 48 h, and subsequently dissociated with trypsin and prepared for analysis by flow cytometry.

### Flow cytometry and analysis of seeding activity

Biosensor cell lines were harvested with 0.05% trypsin, and quenched with media (DMEM + 50% FBS, 1% Pen/Strep, 1% Glutamax). Cells were spun at 500 × g and resuspended in 2% PFA in 1× PBS. Cells were subsequently spun and resuspended in flow buffer (HBSS + 1% FBS + 1 mM EDTA) and stored for less than 24 h prior to performing flow cytometry. Flow cytometry for all samples was performed using a BD Biosciences LSR Fortessa. Flow cytometry data were analyzed as previously described [33]. Seeding activity was calculated as (percentage of FRET-positive cells) × (median fluorescence intensity), which was normalized to negative control samples (tau KO mouse brain).

### Semiquantitative tau histopathology analysis

Individual microscopic slides from each case were staged for AD-associated lesions by H.B. prior to decoding and analysis of the corresponding punch biopsies made from adjacent unstained tissue sections (S.K., T.T.). The LC, TRE/EC, STG, and primary VC were assessed as follows: 0 = no detectable AT8-immunoreactivity, (+) = at least one AT8-immunopositive axon and/or cell soma, + = mild AT8-immunopositive pathology, ++ = moderate AT8-immunoreactive pathology, +++ = severe AT8-immunoreactive pathology. AGD was assessed as follows: 0 = no detectable AT8-immunoreactivity, 1 = mild pathology, 2 = moderate pathology, 3 = severe pathology.

### Statistical analyses

All samples collected by punch biopsy in Ulm were blinded to neuropathological stage prior to performing seeding assay analyses in the Diamond laboratory. All samples from an individual brain region were assessed in parallel with tau KO mouse brain samples. A stringent seeding threshold was set at 4 standard deviations (SD) above the average signal obtained from negative control tau KO mouse brain samples. Flow cytometry gating and analysis of seeding activity were completed prior to the decoding and interpretation of seeding results. All statistical analysis was performed using GraphPad Prism. Kruskal–Wallis one-way analysis of variance (ANOVA) with Dunn’s multiple comparisons test was performed to compare seeding between tau stages and control tau KO samples within each brain region. A K-W ANOVA was also performed to compare PART and AD subjects at NFT I–IV for each brain region. The TRE/EC and LC were directly compared to tau KO control samples by K-W ANOVA. Spearman *r* correlation was calculated for correlation of seeding activity between each brain region.

## Results

### Reproducible seeding activity in adjacent sections

**Fig. 1:**
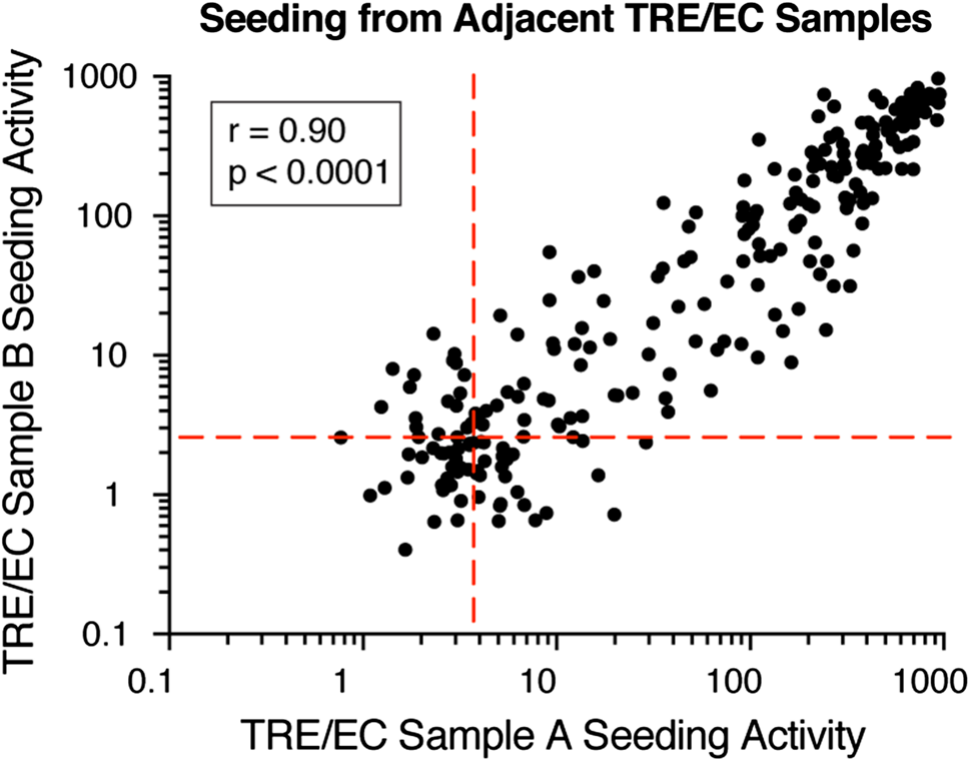
Tau seeding assay reliably detects tau aggregate pathology in formaldehyde-fixed tissue from neuropathologically staged cases. Seeding activity from adjacent punch biopsies correlated significantly with one another (*n* = 247, *p* < 0.0001). Two adjacent punch biopsies were taken from the TRE/EC region and tested for phospho-tau seeding activity. Seeding activity correlated well between punches. Spearman *r* and *p* values are displayed on the graph

We previously developed a protocol to compare seeding activity from fixed brain section punch biopsies in mice with AT8 immunostaining in adjacent tissue sections [29]. To verify the reliability of this method in the human brain, we compared seeding activity in two adjacent 4 mm punch biopsies taken from the combined TRE/EC region in individual AD and putative PART fixed brain samples. We homogenized samples using sonication, and transduced lysate into previously described biosensor cells [24]. We then quantified tau seeding based on the degree of intracellular aggregation measured by FRET flow cytometry, relative to brain samples from tau KO mice [24, 29]. In these studies, we set a highly stringent threshold of 4 SD over background as a “positive” signal. In the present study, we observed good correlation between adjacent punch biopsy samples (*n* = 247 cases, *r* = 0.90, *p* < 0.0001) (Fig. 1).

### Seeding increases with higher tau stages in AD and PART

**Fig. 2:**
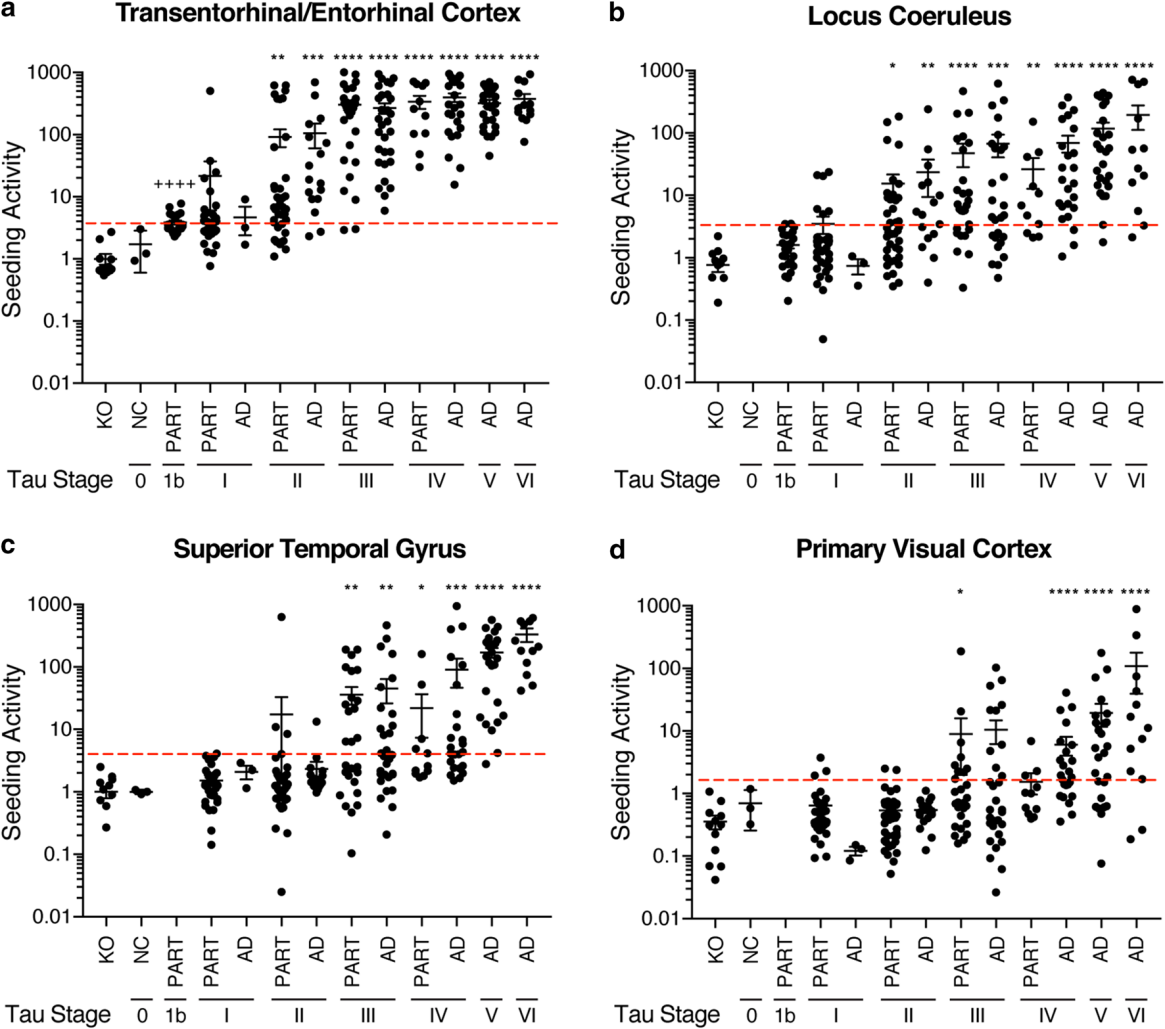
Tau seeding activity across brain regions. Tau seeding activity and tau staging was performed blinded for each of four brain regions in *n* = 247 subjects: TRE/EC, LC, STG (Brodmann Area 22), and primary VC (Brodmann Area 17, striate area). For cortical pretangle stage 1b, samples were taken only from the LC and TRE/EC. **a** Seeding activity was first observed in the TRE/EC at stage 1b, and increased several-fold at later NFT stages. Every individual examined showed positivity in this region by NFT stage IV. **b** Seeding in the LC was first detectable at NFT stage I in a small number of cases. Most samples exhibited tau seeding by NFT stage III. **c** Seeding activity in the STG was observed in a limited number of cases by NFT stage II and increased at later stages. **d** The primary VC displayed positive seeding activity as early as NFT stage III, but approximately 15% of the individuals sampled did not show positivity even at NFT stage VI. KO = tau knockout mouse brain. Threshold values were set at 4SD above tau KO negative control samples. Kruskal-Wallis one-way ANOVA comparing TRE/EC tau stage 1b vs tau KO controls and LC stage 1b vs tau KO controls demonstrated significant seeding activity only in the TRE/EC (++++ = *p* < 0.0001). Additional Kruskal-Wallis ANOVA analysis was performed by comparing tau stages within each brain region to tau KO controls. Error bars = S.E.M, *p < 0.05, **p < 0.01, ***p < 0.001, ****p < 0.0001

Next we assessed seeding activity in a blinded fashion at progressive tau stages. We compared cases classified as the recently defined “definite primary age-related tauopathy” (PART, cortical pretangle stage 1b and NFT stages I–IV, Aβ phase 0) with AD cases (AD, NFT stages I–VI, Aβ phase ≥ 1). 29% of stage 1b subjects and over 50% of NFT stage I subjects displayed seeding activity in the TRE/EC punches (Fig. 2a). In contrast, at stage 1b, a mild AT8 signal was present in the LC and in single or a few pyramidal cells in the TRE. In the LC biopsy punches, 8% of subjects displayed a small degree of tau seeding activity (Fig. 2b). The TRE/EC demonstrated significant seeding activity compared to tau KO control samples at stage 1b, whereas the LC did not display significant seeding activity at this stage (*p* < 0.0001 for TRE/EC, ns for LC). Further, we detected robust seeding activity in the TRE/EC in over 90% of subjects at NFT stage II or higher (Fig. 2b). Seeding in this brain region peaked by NFT stage IV and remained high in later disease stages (Fig. 2b).

When comparing all tau stages to tauKO control samples for each brain region, the TRE/EC and LC displayed significant seeding activity prior to the STG and primary VC. However, in the STG, where AT8 pathology in cortical projection neurons does not develop until NFT stage V, we detected seeding activity at NFT stage III in over 50% of individuals (Fig. 2c). Similarly, 33% of subjects exhibited seeding activity in the primary VC as early as NFT stage III, although AT8 pathology in cortical nerve cells typically develops in this brain region only during the latest stages of AD (Fig. 2d). Seeding in these regions was significantly above tau KO samples as early as tau stage III. Thus, the seeding assay detects tau pathology prior to that which can be visualized by AT8 IHC in brain regions, such as the STG and primary VC. Further, our data are inconsistent with the LC as the origin of seeding in AD and PART, as the TRE/EC shows significant seeding prior to the LC, which did not exhibit robust and consistent seeding activity until NFT stages III–VI.

Notably, we detected no difference in tau seeding activity in the TRE/EC between AD and putative PART subjects at NFT stages I–IV (*p* > 0.05, one-way ANOVA). As for AD, PART subjects also displayed positive seeding activity in brain regions, such as the superior temporal gyrus and primary visual cortex in NFT stages II, III, and IV, despite the absence of AT8-positive NFT pathology. PART and AD exhibited similar overall patterns of progression and levels of tau seeding activity despite the differences in Aβ pathology.

We examined seeding activity in samples that contained concomitant argyrophilic grain disease (AGD), Lewy pathology, or both (Suppl. Fig. 3a–d, Suppl. Tables 3 and 4). Tau seeding was robustly positive in the TRE/EC of cases with concomitant AGD pathology in both PART and AD cases (Suppl. Fig. 3a). This trend was not observed in other brain regions (Suppl. Fig. 3b–d). Coincident Lewy pathology did not appear to enhance tau seeding activity.

### Tau seeding vs. AT8 histopathology

**Fig. 3:**
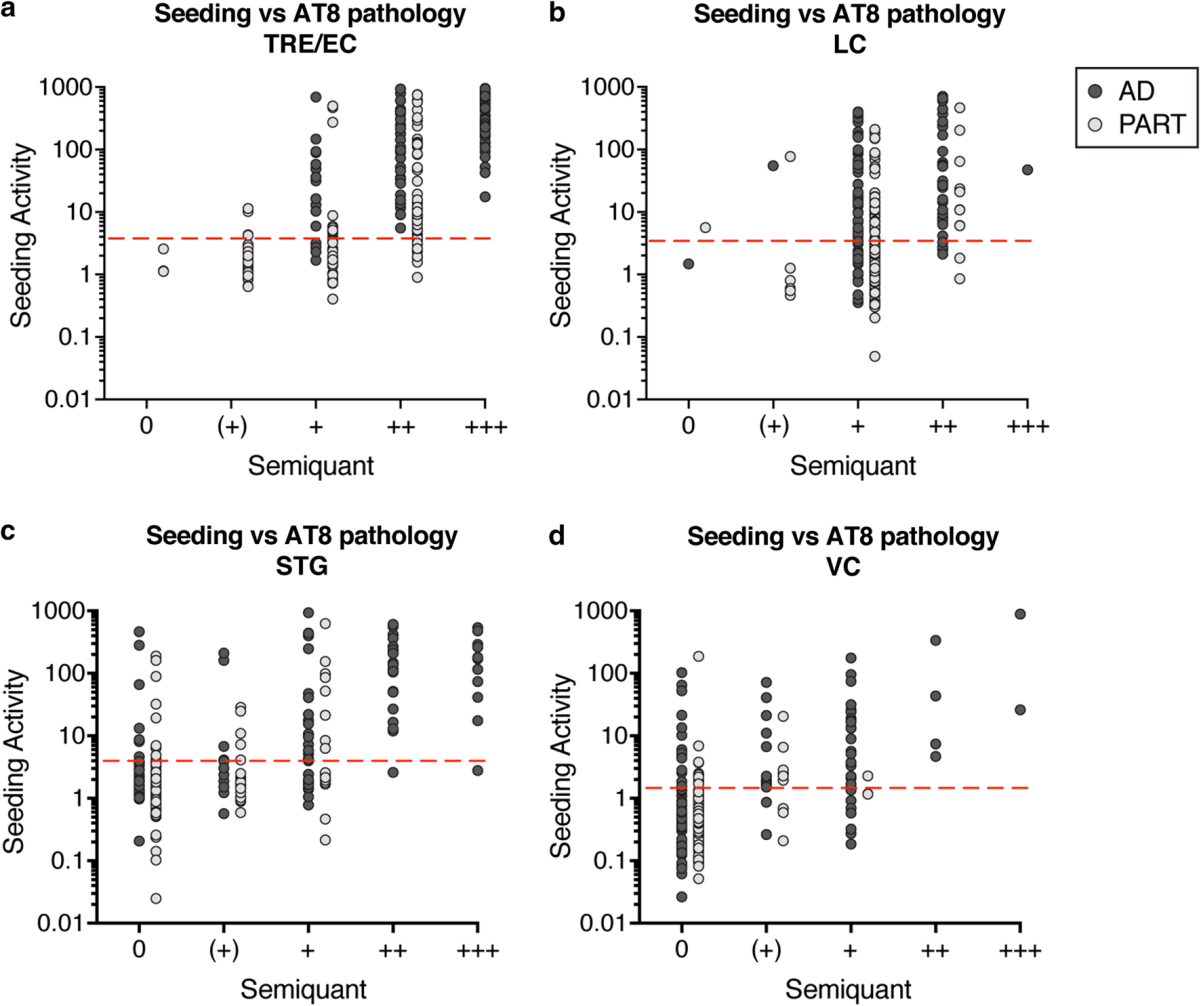
Tau seeding activity versus semiquantitative AT8 histopathology. Seeding activity and AT8 histopathology were each performed blinded, and the results compared. **a** Subjects with a range of AT8 tau pathology (0, (+), +, ++, +++) displayed robust seeding activity in the TRE/EC. Subjects with a higher degree of AT8 signal displayed higher levels of seeding activity. **b** Tau seeding activity in the LC was compared to AT8 signal. Subjects with mild to moderate tau AT8 pathology (levels (+) to ++) had a range of seeding activities, and a substantive number exhibited no seeding activity despite AT8 signal. **c** Seeding in the STG was detectable prior to AT8 pathology in several AD and PART brain samples. **d** Seeding in the primary VC could be detected prior to an AT8 signal in multiple AD and PART brain samples. Note: PART subjects only spanned NFT stages 1b-IV

NFT staging is performed by determining the presence of an AT8 signal across multiple brain regions [8, 25], but direct comparison between AT8 IHC and tau seeding in AD required blinded analysis of an AT8 signal in individual brain regions. Thus, we used AT8 to stain 100 µm brain sections immediately adjacent to those used for the seeding assay. We scored AT8-positive phospho-tau pathology on a semiquantitative scale (see “Methods” section). We then plotted seeding activity against the assessment of AT8-positive IHC in the LC, TRE/EC, STG, and primary VC (Fig. 3a–d). We observed AT8-positive pathology in the absence of detectable seeding, to some extent in the TRE/EC (Fig. 3a), and particularly in the LC (Fig. 3b). We also observed tau seeding in the absence of an AT8 signal, most notably in the STG and primary VC (Fig. 3c, d). However, the vast majority of samples with strong AT8-positive pathology also displayed robust seeding activity. These data were consistent with our prior observation that tau seeding anticipates AT8 IHC in cortical regions that typically score positive at late NFT stages [19].

### TRE/EC seeding precedes tau pathology in other brain regions

**Fig. 4:**
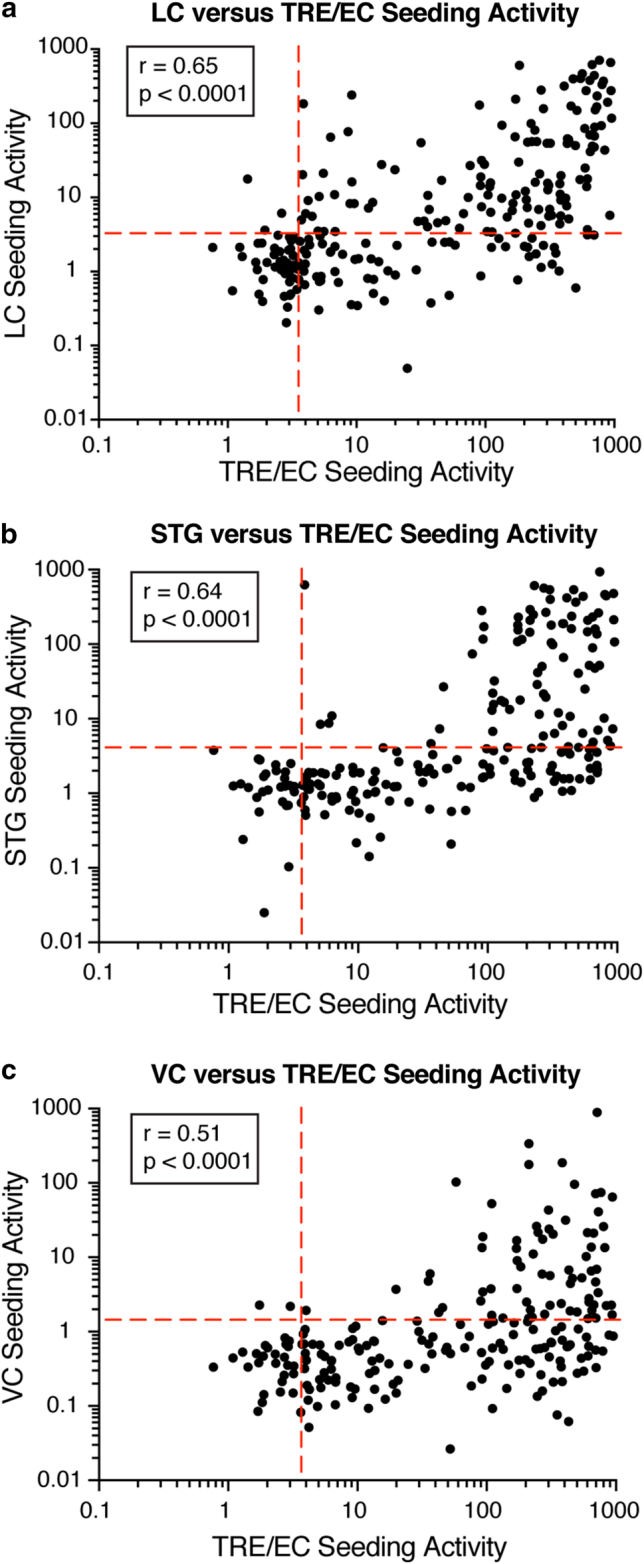
Correlations of tau seeding activity across brain regions. **a** Tau seeding activity was typically observed in the TRE/EC before seeding in the LC and was higher in this brain region for the majority of subjects. Spearman *r* and *p* values are displayed on the graph. **b** Seeding typically appeared first in the TRE/EC and at higher levels than in the STG or **c** the primary VC. Spearman *r* and *p* values are displayed on the graph

To further evaluate the pattern of progression of aggregated tau in different brain regions, we correlated tau seeding between the TRE/EC and other brain regions for individual subjects (Fig. 4). The TRE/EC exhibited seeding activity when other regions did not, consistent with the idea that the TRE/EC rather than the LC is the first region to develop pathogenic forms of tau. When seeding activity was compared between the LC, STG, and primary VC, we observed a hierarchical pattern, with seeding developing in the LC and FTG without a strong AT8 signal in the VC (Suppl. Fig. 4a–c).

### Accumulation of tau seeding within subjects

**Fig. 5:**
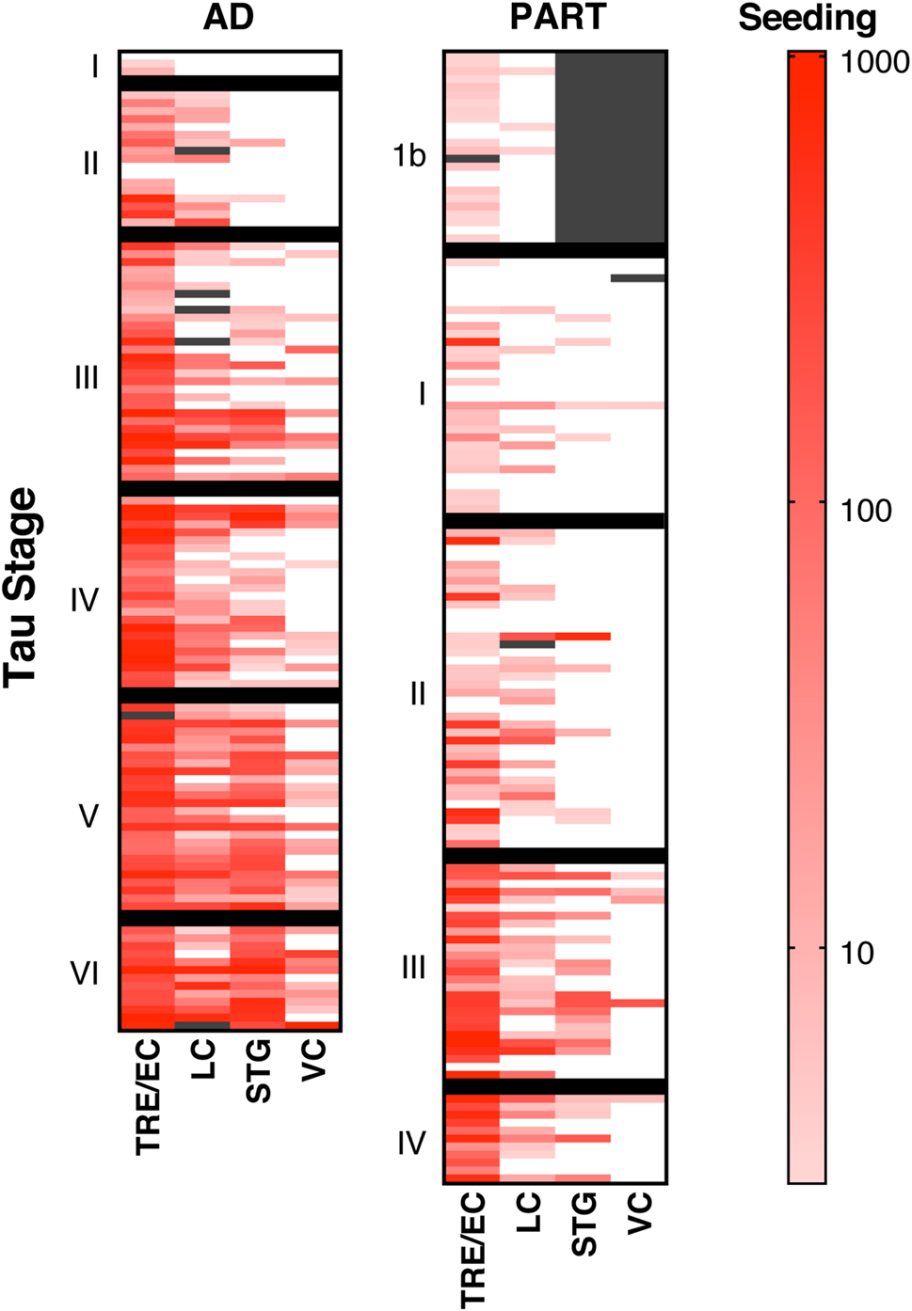
Tau seeding activity across multiple brain regions for individual AD and PART cases. Cases were categorized as AD vs. PART based on neuropathological criteria. Samples from each individual were directly compared across multiple brain regions. A continuous heat map of tau seeding activity (logarithmic scale) was plotted for each case and organized by staging and disease entity (AD, PART). AD subjects were arranged within each stage from low to high Aβ. Gray boxes represent unavailable samples. Tau seeding in the TRE/EC increased first and remained high for each disease stage. Subjects typically displayed less seeding in the LC, STG and primary VC vs. the TRE/EC. The level of tau seeding in these secondary brain regions was higher at later tau stages. Cases categorized as “definite PART” (Aβ phase 0) displayed a similar trend for the spatiotemporal progression of seeding activity when compared to AD. Grey boxes indicate absent samples

To examine the progression of tau pathology across all brain regions, we created a heat map of tau seeding activity for each subject studied (Fig. 5). The TRE/EC reliably developed seeding first in AD and PART cases, and seeding intensity increased in all subjects at later NFT stages. Moreover, we observed a clear hierarchy within individual subjects, with the highest seeding typically appearing in the TRE/EC. We saw no consistent increase in seeding within the LC until NFT stage III. In contrast, we consistently observed early stage seeding in the TRE/EC and late stage increases in seeding in the STG. Several PART subjects also displayed seeding activity in the primary VC as early as NFT stage III, and we observed a clear gradient of seeding activity across individual subjects at increasing stages. Despite a similar pattern and degree of seeding in AD and PART, a larger number of AD subjects had robust seeding in the primary VC at NFT stages III and IV (Fig. 5). However, this difference was not statistically significant at this number of cases (*p* > 0.05, one-way ANOVA).

## Discussion

To test fundamental ideas about AD and PART, we have used a highly sensitive and specific tau biosensor assay to measure seeding activity quantitatively in formaldehyde-fixed brain tissues ~ 100 µm from adjacent sections staged by classical AT8 IHC. There has previously been uncertainty about the origin of AD pathology and whether it arises in the LC or the TRE/EC. Similarly, it remains unclear whether AD and PART constitute distinct neuropathological processes or are variants of the same disorder. Finally, it has not been definitively tested whether tau seeding activity in human brain anticipates subsequent NFT pathology, as would be predicted by the prion model.

Recent work proposes that AD and PART may be different diseases [11]. PART cases are defined as having minimal Aβ pathology or lacking it entirely, and generally feature a relatively limited spread of tau pathology into cortical regions beyond the TRE/EC and hippocampus [11]. In this study, we did not observe a pattern of tau histopathology in AD (i.e., with coincident Aβ pathology, *n* = 113) that was clearly distinct from cases considered to represent “definite PART” (Aβ phase 0, *n* = 134), and seeding activity was similar in both groups across the TRE/EC, LC, STG, and primary VC. With one exception (primary visual cortex at NFT stage IV), we observed a similar pattern of progression and seeding activity for both groups across all neuropathological stages despite different levels of Aβ deposition. This contrasts with a recent report of higher seeding activity in the presence of plaque pathology [3]. This may reflect that we sampled identical regions from the same fixed tissue block (separated by ~ 100 µm) instead of separate fresh and formaldehyde-fixed tissues, and that we evaluated a larger number of cases (*n* = 247 vs. *n* = 11). It remains unknown whether PART and AD might arise from distinct tau prion strains. Future work that examines the tau seed conformations (i.e., strains) present in these cases will help elucidate whether PART constitutes a separate disease entity [11] or represents a prodromal form of AD [14, 15].

Despite the early AT8-positive signal, we typically observed tau seeding activity in the LC only after it was already prominent in the TRE/EC, i.e., at later NFT stages (IV–VI). This is not consistent with the LC as the origin of tau seeding pathology. Instead, our data are consistent with the idea that tau seeds spread from the TRE/EC to the LC and then to more distant cortical regions, such as the STG and, subsequently, the primary VC.

We have attempted to combine two orthogonal measures of pathology: classical IHC and a cell-based assay that depends on detection of bioactive tau seeding activity. Seeding and phospho-tau pathology did not uniformly correlate. For example, we observed AT8-positivity in the LC in the absence of seeding activity and seeding activity in the STG and primary VC in the absence of clear NFT pathology. In this study, we only examined tau seeding activity in brain regions that had been previously described to accumulate phospho-tau pathology by AT8 IHC, and thus were biased towards brain regions “classically” affected with NFTs. Indeed, other brain regions may also show seeding activity in the absence of AT8-positivity. To fully understand the relationship of seeding to pathology in AD, testing of multiple brain regions across NFT stages will be required. Interestingly, we note that in our prior study of tau seeding activity in fresh frozen tissue of subjects with AD, we observed seeding activity in the cerebellum of 3/6 subjects with late stage AD (a region that virtually never shows overt NFT pathology) [19].

In testing various ideas about the origins and progression of AD and PART, this work is the first to combine a bioassay of tau seeding activity directly with classical histopathology on adjacent, formaldehyde-fixed tissue sections. We observed no discernible differences between AD and PART with regard to AT8 immunostaining at NFT stages I–IV. We also found no evidence to support the idea that an early AT8 signal in the LC indicates that this region is the initial source of pathogenic seeding in AD. Instead, our data are consistent with the TRE/EC as the first site that develops tau seeding activity. Finally, we clearly observed that tau seeding activity anticipates detectable NFT pathology in the STG and primary VC, which is consistent with the prion model of transcellular propagation of tau seeds as a driver of disease progression.

## Electronic supplementary material

Below is the link to the electronic supplementary material.

Supplemental Figure 1. Summary of staging for AT8-positive tau pathology in AD. **a** Diagrams of brain regions of interest for AT8 phospho-tau pathology observed at different tau stages. **b** Stage 1b was the earliest examined. Phospho-tau pathology was observed in the LC, and very limited AT8 pathology was present in the TRE. **c** NFT stages I–VI include increasing levels of phospho-tau pathology in specific brain regions. NFT stage I includes TRE pathology. This includes the EC by NFT stage II. Stage III includes pathology in the hippocampus. Stage IV includes pathology in the middle temporal gyrus and insula. Stage V includes pathology in additional cortical regions, including the superior temporal gyrus (STG, Brodmann Area 22). However, only NFT stage VI includes tau pathology in the primary visual cortex (VC, Brodmann Area 17, striate area). AT8 pathology is represented by red dots. (TIFF 5711 kb)

Supplemental Figure 2. Sites of 4 mm biopsy punches in 100 µm unstained formaldehyde-fixed tissue brain sections. **a** Coronal hemisphere section through the anteromedial temporal lobe containing uncal portions of the hippocampus, the parahippocampal gyrus, and adjoining temporal gyri. **b, d** Detailed views of **a** showing the regions (TRE, EC, STG) where punches were made (examples of punch locations are marked by blue dots). **c** Coronal hemisphere section through the basal occipital lobe, including the lower bank of the calcarine fissue together with portions of the peristriate, parastriate, and striate areas (Brodmann Areas 17–19). **f** Detailed view of **c** showing BA 17-19 (adjacent punches, here in blue, were made only in the striate area (BA 17). **e** Detailed view of the pontine tegmentum containing the locus coeruleus (LC). Punches were made on both sides of the same section. Abbreviations: BA – Brodmann Area; CA 1 – Ammon’s horn, first sector; parasubic. – parasubiculum, transentorhin. – transentorhinal. (TIFF 8713 kb)

Supplemental Figure 3. Tau seeding in cases with coincident argyrophilic grain disease (AGD) (*n* = 18), Lewy pathology (Syn) (*n* = 18), or both (*n* = 2). **a** Tau seeding activity in the TRE/EC is displayed for subjects with coincident AGD (blue), Lewy pathology (red), or both (blue/red). Tau seeding activity in the TRE/EC is robust in cases with coincident AGD pathology. **b** Tau seeding activity in the LC. **c** Tau seeding activity in the STG. **d** Tau seeding activity in the primary VC. (TIFF 7115 kb)

Supplemental Figure 4. Correlation of tau seeding activity between the LC, STG, and primary VC. **a** Tau seeding in the LC was typically higher than in the STG. However, several subjects displayed the opposite trend, with STG showing seeding with no seeding in the LC. Spearman r and p values are displayed on the graph. **b** The LC typically displayed higher seeding than the primary VC. Spearman r and p values are displayed on the graph. **c** Seeding activity in the STG was typically higher than in the primary VC. Spearman r and p values are displayed on the graph. (TIFF 4333 kb)

Supplemental Table 1. Summary of AD-related neurofibrillary tangle (NFT) stages (DOCX 12 kb)

Supplemental Table 2. Summary of control samples (DOCX 14 kb)

Supplemental Table 3. Summary of AGD and α-synuclein pathology in AD cases (DOCX 14 kb)

Supplemental Table 4. Summary of AGD and α-synuclein pathology in PART cases (DOCX 13 kb)
