# Supplementary figures and images for: Tau seeding activity begins in the transentorhinal/entorhinal regions and anticipates phospho-tau pathology in Alzheimer’s disease and PART
Source: Acta Neuropathol. 2018 May 11;136(1):57–67. doi: 10.1007/s00401-018-1855-6 (PMC6015098; doi:10.1007/s00401-018-1855-6)

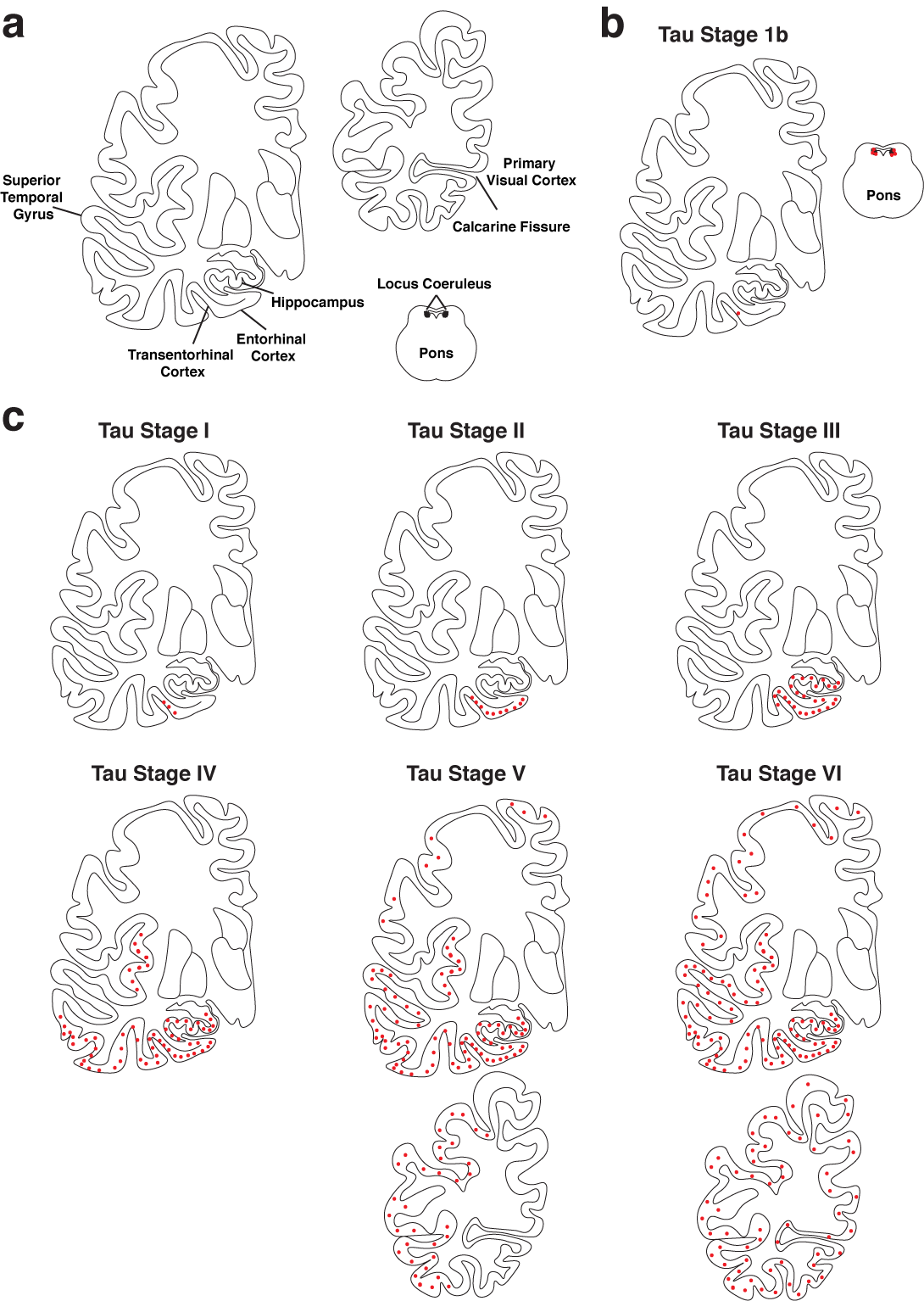

Supplement: Supplemental Figure 1. Summary of staging for AT8-positive tau pathology in AD. — a Diagrams of brain regions of interest for AT8 phospho-tau pathology observed at different tau stages. b Stage 1b was the earliest examined. Phospho-tau pathology was observed in the LC, and very limited AT8 pathology was present in the TRE. c NFT stages I–VI include increasing levels of phospho-tau pathology in specific brain regions. NFT stage I includes TRE pathology. This includes the EC by NFT stage II. Stage III includes pathology in the hippocampus. Stage IV includes pathology in the middle temporal gyrus and insula. Stage V includes pathology in additional cortical regions, including the superior temporal gyrus (STG, Brodmann Area 22). However, only NFT stage VI includes tau pathology in the primary visual cortex (VC, Brodmann Area 17, striate area). AT8 pathology is represented by red dots. (TIFF 5711 kb) [file 401_2018_1855_MOESM1_ESM.tif]

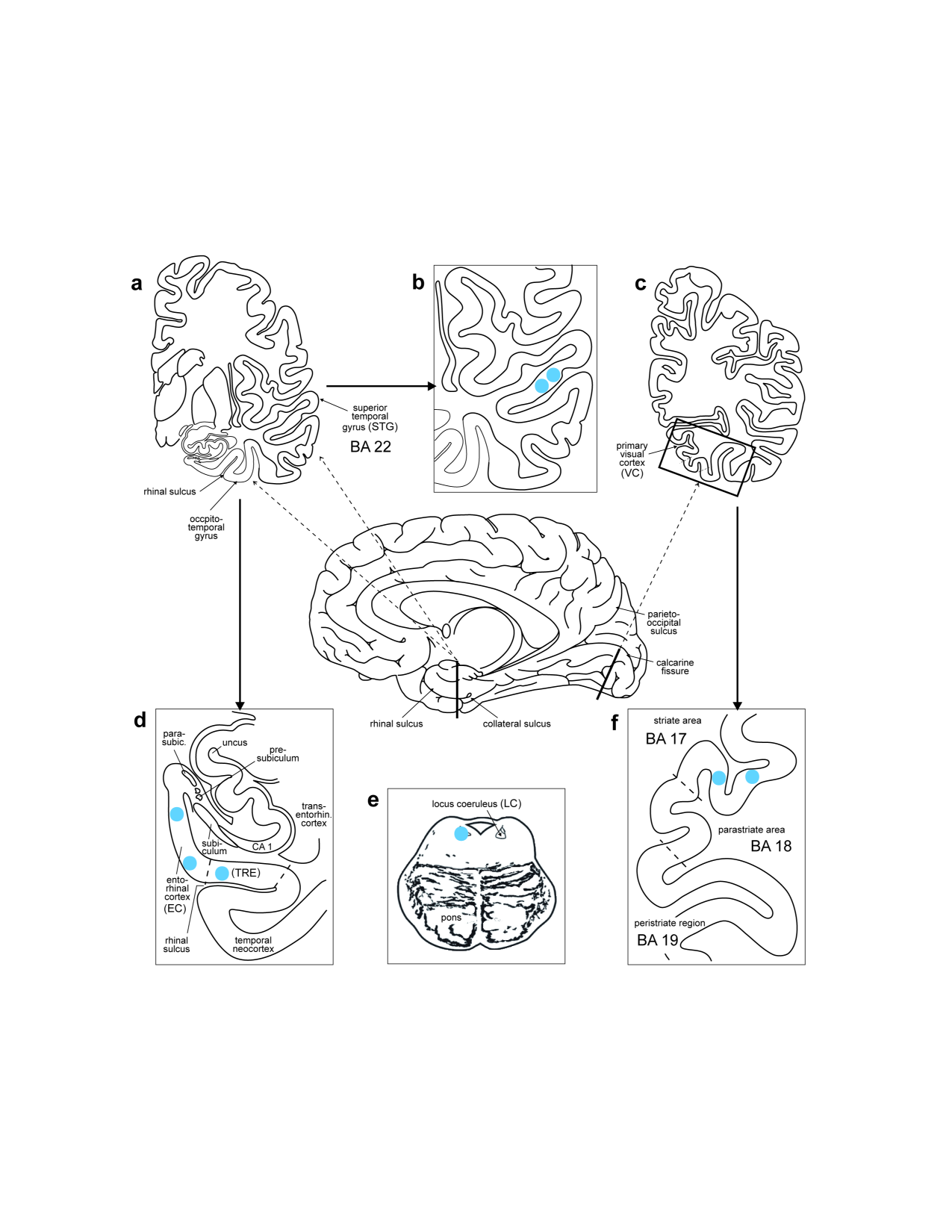

Supplement: Supplemental Figure 2. Sites of 4 mm biopsy punches in 100 µm unstained formaldehyde-fixed tissue brain sections. — a Coronal hemisphere section through the anteromedial temporal lobe containing uncal portions of the hippocampus, the parahippocampal gyrus, and adjoining temporal gyri. b, d Detailed views of a showing the regions (TRE, EC, STG) where punches were made (examples of punch locations are marked by blue dots). c Coronal hemisphere section through the basal occipital lobe, including the lower bank of the calcarine fissue together with portions of the peristriate, parastriate, and striate areas (Brodmann Areas 17–19). f Detailed view of c showing BA 17-19 (adjacent punches, here in blue, were made only in the striate area (BA 17). e Detailed view of the pontine tegmentum containing the locus coeruleus (LC). Punches were made on both sides of the same section. Abbreviations: BA – Brodmann Area; CA 1 – Ammon’s horn, first sector; parasubic. – parasubiculum, transentorhin. – transentorhinal. (TIFF 8713 kb) [file 401_2018_1855_MOESM2_ESM.tif]

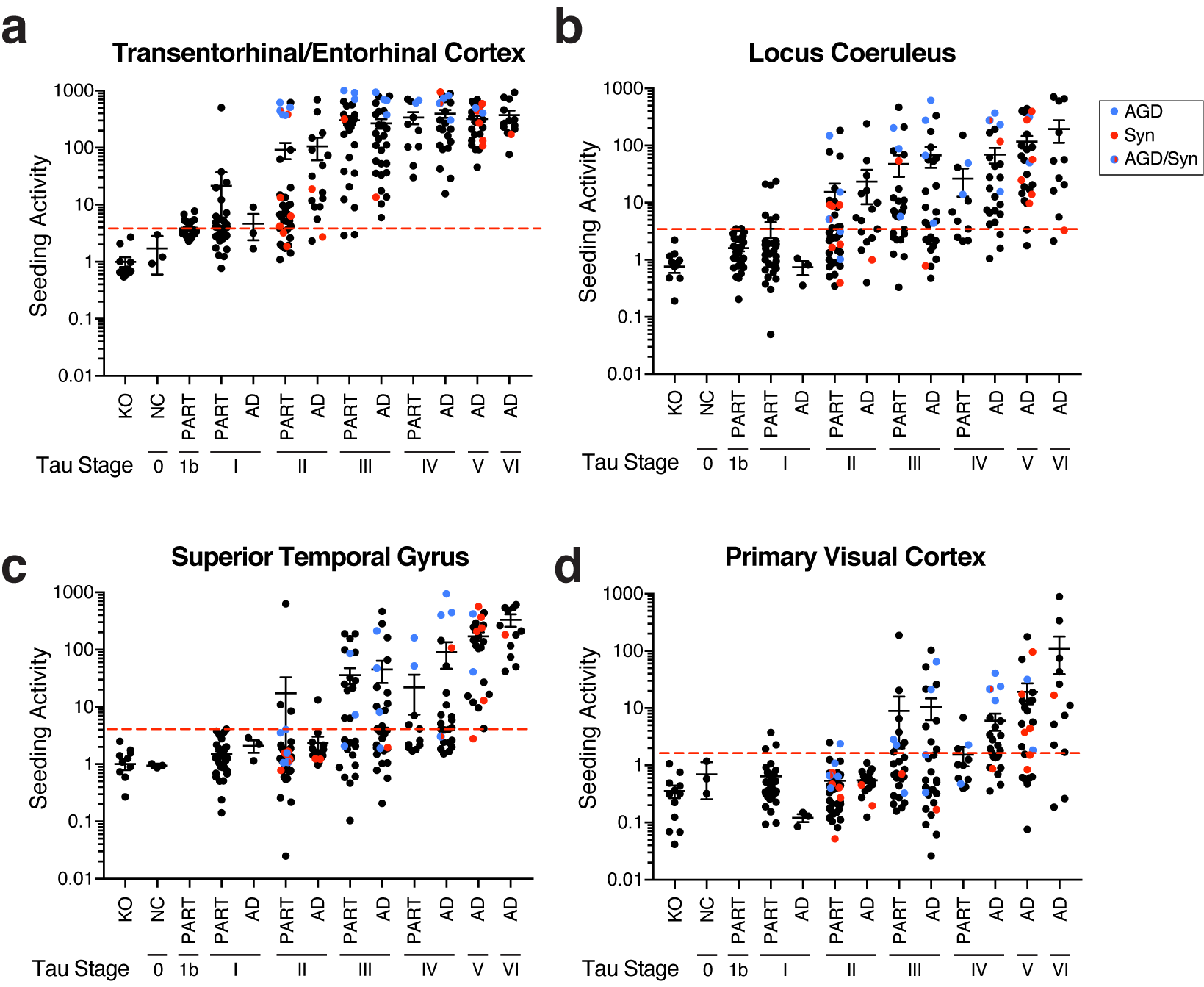

Supplement: Supplemental Figure 3. Tau seeding in cases with coincident argyrophilic grain disease (AGD) (n = 18), Lewy pathology (Syn) (n = 18), or both (n = 2). — a Tau seeding activity in the TRE/EC is displayed for subjects with coincident AGD (blue), Lewy pathology (red), or both (blue/red). Tau seeding activity in the TRE/EC is robust in cases with coincident AGD pathology. b Tau seeding activity in the LC. c Tau seeding activity in the STG. d Tau seeding activity in the primary VC. (TIFF 7115 kb) [file 401_2018_1855_MOESM3_ESM.tif]

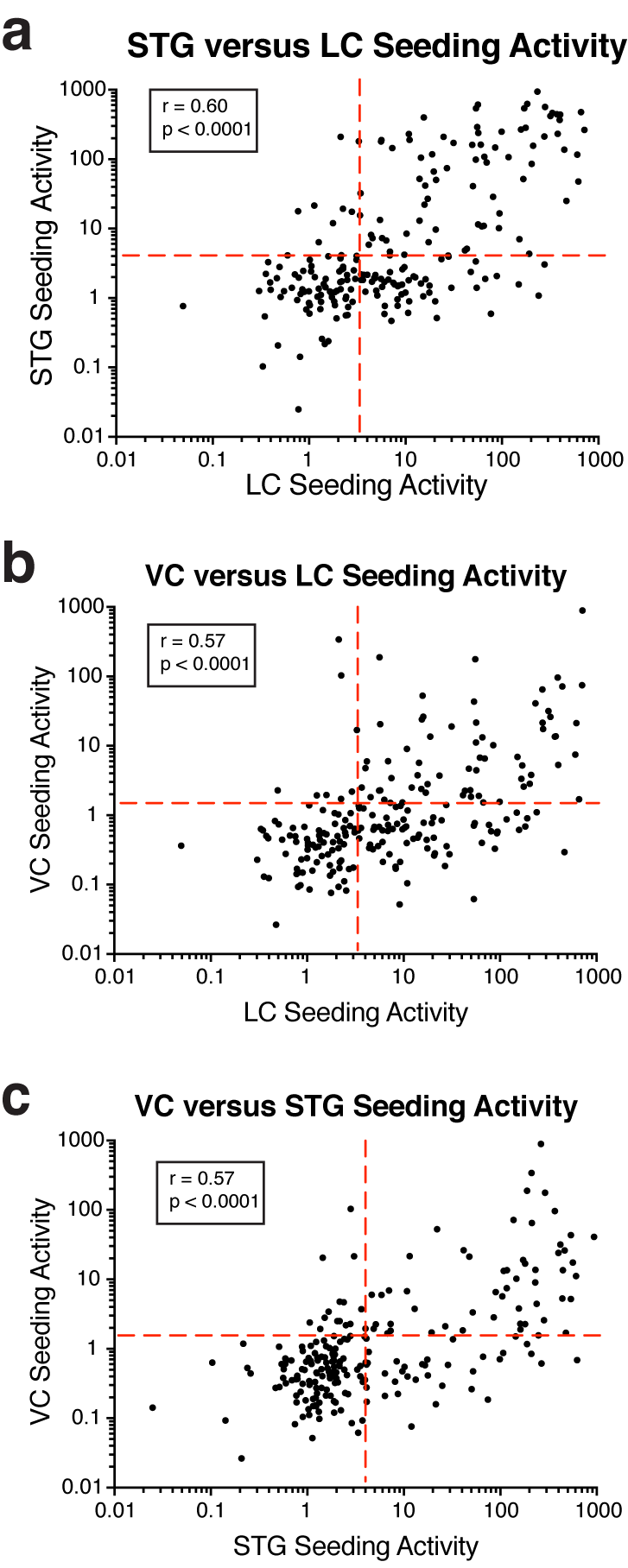

Supplement: Supplemental Figure 4. Correlation of tau seeding activity between the LC, STG, and primary VC. — a Tau seeding in the LC was typically higher than in the STG. However, several subjects displayed the opposite trend, with STG showing seeding with no seeding in the LC. Spearman r and p values are displayed on the graph. b The LC typically displayed higher seeding than the primary VC. Spearman r and p values are displayed on the graph. c Seeding activity in the STG was typically higher than in the primary VC. Spearman r and p values are displayed on the graph. (TIFF 4333 kb) [file 401_2018_1855_MOESM4_ESM.tif]
