# Supplementary material for: Tau seeding activity begins in the transentorhinal/entorhinal regions and anticipates phospho-tau pathology in Alzheimer’s disease and PART
Source: Acta Neuropathol. 2018 May 11;136(1):57–67. doi: 10.1007/s00401-018-1855-6 (PMC6015098; doi:10.1007/s00401-018-1855-6)
Supplement: Supplemental Table 1. Summary of AD-related neurofibrillary tangle (NFT) stages — (DOCX 12 kb) [file 401_2018_1855_MOESM5_ESM.docx]

**Supplemental Table 1.** **Summary of AD-related neurofibrillary tangle (NFT) stages**

| **NFT I** | Gallyas silver staining reveals neurofibrillary lesions restricted to selected brainstem nuclei and the transentorhinal region (TRE). |
| --- | --- |
| **NFT II** | Tau pathology is present in the entorhinal cortex (EC) of the parahippocampal gyrus. |
| **NFT III** | Tau pathology in the CA1 sector of the hippocampal formation, and in neocortical regions of the temporal neocortex adjoining the TRE. |
| **NFT IV and V** | Increasingly prominent tau pathology in neocortical regions. The superior temporal gyrus (STG, Brodmann Area 22) becomes involved at NFT stage V. |
| **NFT VI** | Tau pathology is present in neocortical areas, such as the primary visual cortex (VC, Brodmann Area 17). |
