# Supplementary material for: Tau seeding activity begins in the transentorhinal/entorhinal regions and anticipates phospho-tau pathology in Alzheimer’s disease and PART
Source: Acta Neuropathol. 2018 May 11;136(1):57–67. doi: 10.1007/s00401-018-1855-6 (PMC6015098; doi:10.1007/s00401-018-1855-6)
Supplement: Supplemental Table 2. Summary of control samples — (DOCX 14 kb) [file 401_2018_1855_MOESM6_ESM.docx]

| **Blinded controls** | **AGD** | **Age** | **m/f** |
| --- | --- | --- | --- |
| Negative | 0 | 50 | m |
| Negative | 0 | 45 | f |
| Negative | 0 | 49 | f |
| Positive (NFT I) | 0 | 50 | m |
| Positive (NFT I) | 0 | 59 | f |
|  |  |  |  |
| **Unblinded control** | **AGD** | **Age** | **m/f** |
| Positive (NFT VI) | 0 | 72 | f |

**Supplemental Table 2.** **Summary of Control Samples**
