# Supplementary material for: Tau seeding activity begins in the transentorhinal/entorhinal regions and anticipates phospho-tau pathology in Alzheimer’s disease and PART
Source: Acta Neuropathol. 2018 May 11;136(1):57–67. doi: 10.1007/s00401-018-1855-6 (PMC6015098; doi:10.1007/s00401-018-1855-6)
Supplement: Supplemental Table 3. Summary of AGD and α-synuclein pathology in AD cases — (DOCX 14 kb) [file 401_2018_1855_MOESM7_ESM.docx]

**Supplemental Table 3.** **Summary of AGD and α-synuclein Pathology in AD Cases**

| **AD** | | | | | |
| --- | --- | --- | --- | --- | --- |
| **Argyrophilic Grain Disease (AGD) Pathology** | | | | | |
| **Tau** | **Abeta** | **AGD** | **Syn** | **Age** | **m/f** |
| III | 1 | 2 | 0 | 75 | m |
| III | 3 | 2 | 0 | 71 | f |
| III | 2 | 3 | 0 | 86 | f |
| III | 3 | 3 | 0 | 71 | f |
| IV | 1 | 1 | 0 | 84 | m |
| IV | 1 | 3 | 0 | 68 | m |
| IV | 1 | 3 | 0 | 70 | m |
| V | 2 | 1 | 0 | 75 | f |
| V | 2 | 2 | 0 | 81 | f |
|  |  |  |  |  |  |
| **Alpha Synuclein Pathology** | | | | | |
| **Tau** | **Abeta** | **AGD** | **Syn** | **Age** | **m/f** |
| II | 2 | 0 | 3 | 69 | m |
| II | 2 | 0 | 4 | 72 | f |
| III | 1 | 0 | 4 | 77 | m |
| IV | 3 | 0 | 2 | 91 | f |
| V | 3 | 0 | 1 | 92 | m |
| V | 3 | 0 | 3 | 93 | f |
| V | 5 | 0 | 4 | 80 | f |
| V | 5 | 0 | 4 | 80 | f |
| V | 4 | 0 | 5 | 76 | m |
| V | 5 | 0 | 5 | 76 | f |
| VI | 3 | 0 | 5 | 71 | m |
|  |  |  |  |  |  |
| **AGD/Synuclein Pathology** | | | | | |
| **Tau** | **Abeta** | **AGD** | **Syn** | **Age** | **m/f** |
| IV | 4 | 2 | 4 | 94 | f |
