# Supplementary material for: Tau seeding activity begins in the transentorhinal/entorhinal regions and anticipates phospho-tau pathology in Alzheimer’s disease and PART
Source: Acta Neuropathol. 2018 May 11;136(1):57–67. doi: 10.1007/s00401-018-1855-6 (PMC6015098; doi:10.1007/s00401-018-1855-6)
Supplement: Supplemental Table 4. Summary of AGD and α-synuclein pathology in PART cases — (DOCX 13 kb) [file 401_2018_1855_MOESM8_ESM.docx]

**Supplemental Table 4.** **Summary of AGD and α-synuclein Pathology in PART Cases**

| **PART** | | | | | |
| --- | --- | --- | --- | --- | --- |
| **Argyrophilic Grain Disease (AGD) Pathology** | | | | | |
| **Tau** | **Abeta** | **AGD** | **Syn** | **Age** | **m/f** |
| II | 0 | 1 | 0 | 71 | m |
| II | 0 | 1 | 0 | 67 | f |
| II | 0 | 1 | 0 | 50 | f |
| II | 0 | 2 | 0 | 57 | m |
| III | 0 | 1 | 0 | 61 | m |
| III | 0 | 3 | 0 | 75 | m |
| III | 0 | 1 | 0 | 77 | m |
| IV | 0 | 1 | 0 | 88 | f |
| IV | 0 | 2 | 0 | 74 | m |
|  |  |  |  |  |  |
| **Alpha Synuclein Pathology** | | | | | |
| **Tau** | **Abeta** | **AGD** | **Syn** | **Age** | **m/f** |
| II | 0 | 0 | 1 | 76 | f |
| II | 0 | 0 | 2 | 50 | f |
| II | 0 | 0 | 2 | 61 | m |
| II | 0 | 0 | 3 | 65 | f |
| II | 0 | 0 | 4 | 76 | m |
| II | 0 | 0 | 4 | 75 | f |
| III | 0 | 0 | 2 | 76 | m |
|  |  |  |  |  |  |
| **AGD/Synuclein Pathology** | | | | | |
| **Tau** | **Abeta** | **AGD** | **Syn** | **Age** | **m/f** |
| II | 0 | 2 | 1 | 68 | m |
